# Mechanical ventilation and *Streptococcus pneumoniae* pneumonia alter mitochondrial homeostasis

**DOI:** 10.1038/s41598-018-30226-x

**Published:** 2018-08-06

**Authors:** Mathieu Blot, Laure-Anne Pauchard, Irène Dunn, Jennifer Donze, Stéphanie Malnuit, Chloé Rebaud, Delphine Croisier, Lionel Piroth, Jérôme Pugin, Pierre-Emmanuel Charles

**Affiliations:** 1 Lipides Nutrition Cancer Lab, U.M.R. 1231, I.N.S.E.R.M., Faculty of Health Sciences, University of Burgundy, Dijon, France; 2 Infectious Diseases Department, Dijon University Hospital, Dijon, France; 3 Intensive Care Laboratory, University Hospitals of Geneva & Faculty of Medicine, University Hospital of Geneva, Geneva, Switzerland; 4 Vivexia S.A.R.L., Gemeaux, France; 5 Intensive Care Unit, Dijon University Hospital, Dijon, France

## Abstract

Required mechanical ventilation (MV) may contribute to bacterial dissemination in patients with *Streptococcus pneumoniae* pneumonia. Significant variations in plasma mitochondrial DNA (mtDNA) have been reported in sepsis according to the outcome. The impact of lung stretch during MV was addressed in a model of pneumonia. Healthy or *S. pneumoniae* infected rabbits were submitted to MV or kept spontaneously breathing (SB). Bacterial burden, cytokines release, mitochondrial DNA levels, integrity and transcription were assessed along with 48-hour mortality. Compared with infected SB rabbits, MV rabbits developed more severe pneumonia with greater concentrations of bacteria in the lungs, higher rates of systemic dissemination, higher levels of circulating inflammatory mediators and decreased survival. Pulmonary mtDNA levels were significantly lower in infected animals as compared to non-infected ones, whenever they were SB or MV. After a significant early drop, circulating mtDNA levels returned to baseline values in the infected SB rabbits, but remained low until death in the MV ones. Whole blood *ex-vivo* stimulation with *Streptococcus pneumoniae* resulted in a reduction of polymorphonuclear leukocytes mitochondrial density and plasma mtDNA concentrations. Thus, persistent mitochondrial depletion and dysfunction in the infected animals submitted to MV could account for their less efficient immune response against *S. pneumoniae*.

## Introduction

Community acquired pneumonia remains the main cause of death from infection, and *Streptococcus pneumoniae* is the main bacterial agent^1^. In addition, in severely ill individuals, mechanical ventilation (MV) may contribute to bacterial dissemination^2,3^. It is likely that the combination of the damaging effects of MV on the lungs (i.e., Ventilator-Induced Lung Injury [VILI], release of pro-inflammatory cytokines, recruitment and activation of polymorphonuclear leukocytes [PMNs]) and the onset of infection influences the severity of the disease^4–7^. This pro-inflammatory state could alter host defenses against bacterial invasion^8–10^. However, the relationship between excessive inflammation and resulting sepsis has been challenged by the concept of immune paralysis^11^.

The inflammatory response to bacteria is initiated by the detection of pathogen-associated molecular patterns (PAMPs) by specialized cognate receptors, including toll-like receptors (TLRs)^12^. Various kinds of molecules released from damaged or dying cells are also likely to activate immunity, and share signaling pathways and patterns of response with PAMPs^13^. Among the so-called damage-associated molecular patterns (DAMPs), some originate from mitochondria^14,15^. Thus, mitochondrial DNA (mtDNA) activates and amplifies inflammation through the TLR9 ligation, as well as through the NLRP3 and the STING pathways^13,16,17^. In addition, the formyl-Met-Leu-Phe (fMLP) peptide directs PMNs towards injured tissues, and might contribute to VILI^18,19^. The extra-cellular release of the so called mitochondrial alarmins is the result of poorly understood mechanisms including extrusion^20^. In addition, it is now well established that the metabolic disturbances subsequent to the activation of immune cells depend heavily on mitochondrial homeostasis, driving their ability to clear microbial invaders^21^. Thus, stressed mitochondria release ROS which likely contribute to the killing of bacteria by activated immune cells. Moreover, mitophagy is enhanced in order to protect the cell against the “toxicity” of such damaged organelles, leading in turn to transient mitochondria depletion, reduction in energy expenditure and inflammation control. However, mitochondria involvement is not well understood in the context of bacterial infection or VILI. Some experimental reports suggest that fMLP might contribute to VILI^18,19^. In contrast, there are conflicting results regarding the link between circulating mtDNA and outcomes of septic patients^22–25^. Finally, mtDNA deletions have been reported in various chronic diseases and could be associated with substantial mitochondrial disturbances^26^. Our investigation was therefore focused on whether MV and *S. pneumoniae* pneumonia could induce quantitative and qualitative mtDNA abnormalities in a rabbit model of infection^20^.

## Results

### Mechanical ventilation worsens both clinical and microbiological outcomes of pneumococcal pneumonia in rabbits

Since uninfected animals subjected to 48 hours of MV were alive with arterial blood gases and lactate levels within the normal range, our MV protocol was considered safe (Supplementary Table S1). However, it is worth noting that MV alone led to mild lung injury (Supplementary Fig. S1).

**Figure 1:**
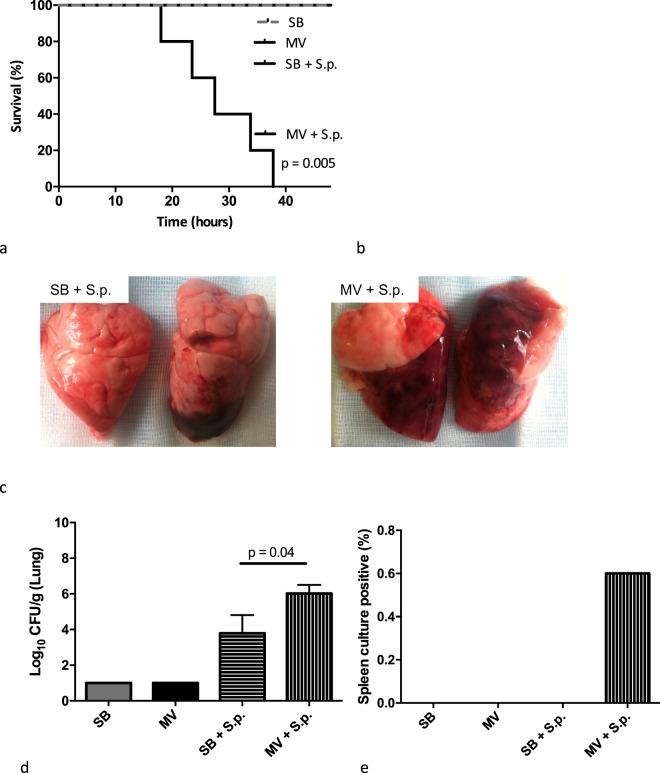
Main features of Streptococcus pneumoniae pneumonia in either spontaneously breathing or mechanically ventilated rabbits. (**a**) Time-dependent probability of survival (log rank test, p = 0.005); (**b**) Lung injury assessment according to macroscopic score calculation; (**c**) Lung pictures after pneumonia in either SB (left) or MV (right) animals; (**d**) pulmonary bacterial concentrations (Log10 CFU per gram of lung); (**e**) Pulmonary-to-systemic bacterial translocation expressed as the spleen positive culture rate 48 hours after bacteria instillation (or at the time of death if earlier). CFU: colony forming unit; MV: mechanical ventilation, SB: spontaneously breathing, S.p.: Streptococcus pneumoniae, IQR: interquartile range.

Forty-eight-hour mortality in infected MV animals was dramatically higher than that in infected SB rabbits (100 vs. 0%, respectively; p = 0.005). Bacterial clearance in the lung was lower (6.0 [5.1–6.5] vs. 3.8 [1.0–4.8]) log_10_CFU/g of lung, p = 0.04), and the rate of systemic dissemination was higher (60 vs. 0%) (Fig. 1). In addition, pneumonia was multifocal in MV animals, whereas it was usually limited to one lobe in their SB counterparts.

Remarkably, MV did not lead to metabolic acidosis in rabbit despite adverse settings (Table S2), whereas such disturbances have been described in smaller animals^27^.

### Mechanical ventilation increases systemic but not pulmonary inflammation in rabbits with pneumonia

**Figure 2:**
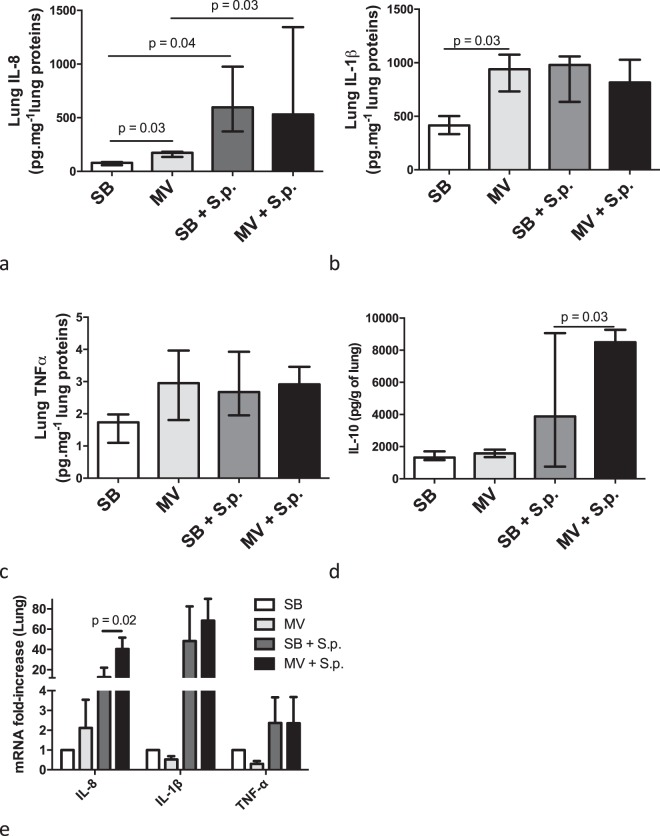
Inflammatory cytokine concentrations and gene expression in the lung tissue of spontaneously breathing or mechanically ventilated rabbits with or without Streptococcus pneumoniae pneumonia. Median (IQR) inflammatory cytokine concentrations (IL-8 [a], IL-1β [b] and TNF-α [c]) in lung homogenates obtained 48 hours (or at the time of death if earlier) after tracheal instillation of saline (controls) or 5.108 CFU of Streptococcus pneumoniae in either spontaneously breathing or mechanically ventilated rabbits. Relative mRNA copies number of IL-8, IL-1β and TNF-α genes within the lung were measured by the reverse transcriptase-polymerase chain reaction. All values are shown as the fold increase compared with uninfected spontaneous breathing rabbits - value set to 1. The Kruskall Wallis test and the Mann-Whitney U test were used successively for all intergroup comparisons and followed by post hoc corrections for multiple comparisons using the Bonferroni method. CFU: colony forming unit; SB: spontaneously breathing, MV: mechanical ventilation, S.p.: Streptococcus pneumoniae, IL: interleukin, TNF: tumor necrosis factor, mRNA: messenger ribonucleic acid, BALF: broncho-alveolar lavage fluid; IQR: interquartile range.

**Figure 3:**
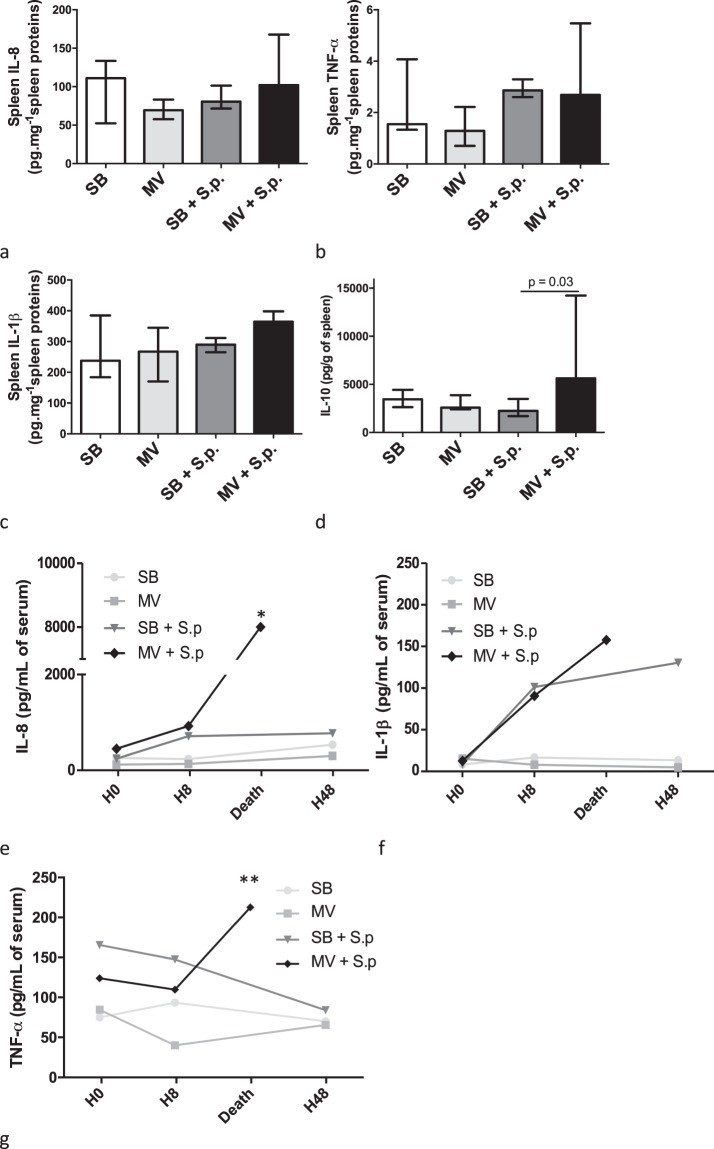
Inflammatory cytokine concentrations and gene expression in the systemic compartment of spontaneously breathing or mechanically ventilated rabbits with or without Streptococcus pneumoniae pneumonia. Median (IQR) inflammatory cytokine concentrations (IL-8 [A], IL-1β [B] and TNF-α [C]) in spleen homogenates, 48 hours (or at the time of death if earlier) after tracheal instillation of saline (controls) or 5.108 CFU of Streptococcus pneumoniae in spontaneously breathing or mechanically ventilated rabbits. Relative mRNA copies numbers of IL-8, IL-1β and TNF-α genes within the spleen were measured by the reverse transcriptase-polymerase chain reaction. All values are shown as the fold increase compared with uninfected spontaneous breathing rabbits - value set to 1. Median plasma concentrations of inflammatory cytokines (IL-8 [e], IL-1β [f] and TNF-α [g]) were measured by ELISA at baseline, 8 or 48 hours (or at the time of death if earlier) after challenge. The Kruskall Wallis test and the Mann-Whitney U test were used successively for all intergroup comparisons and followed by post hoc corrections for multiple comparisons using the Bonferroni method.* and ** denote p < 0.05 between infected SB and MV rabbits at H48 or time of death, respectively. CFU: colony forming unit, SB: spontaneously breathing, MV: mechanical ventilation, S.p.: Streptococcus pneumoniae, IL: interleukin, TNF: tumor necrosis factor, mRNA: messenger ribonucleic acid, BALF: broncho-alveolar lavage fluid; IQR: Interquartile Range.

Pneumonia resulted in increased pulmonary concentrations and gene expression of both IL-8 and IL-1β, but not TNF-α (Fig. 2). However, there was no significant difference between MV and SB infected animals. In contrast, systemic inflammation was enhanced in MV animals according to plasma concentrations of IL-8 and TNF-α, but not IL-1β (Fig. 3). Obviously, the anti-inflammatory cytokine IL-10 was significantly more elevated in the lung as well as in the spleen of the infected rabbits submitted to MV as compared to SB ones.

**Figure 4:**
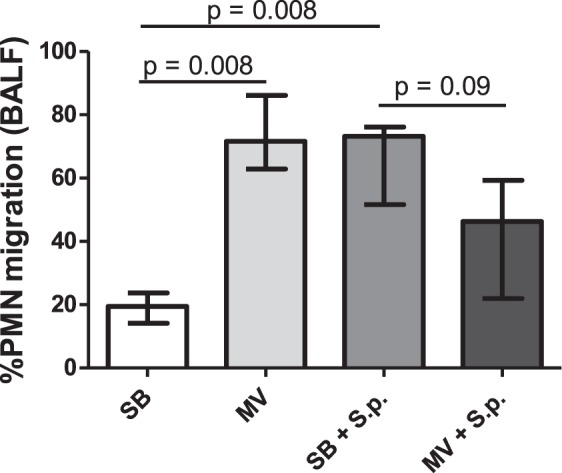
Polymorphonuclear leukocytes chemotaxis induced by broncho-alveolar lavage fluid in spontaneously breathing or mechanically ventilated rabbits with or without Streptococcus pneumoniae pneumonia. PMN chemotaxis was measured using a Boyden chamber, 48 hours (or at the time of death if earlier) after tracheal instillation of saline (controls) or 5.108 CFU of Streptococcus pneumoniae in either spontaneously breathing or mechanically ventilated rabbits. Results are expressed as the mean (SD) % of PMNs from healthy volunteers attracted by rabbit BALF. The Kruskall Wallis test and the Mann-Whitney U test were used successively for all intergroup comparisons and followed by post hoc corrections for multiple comparisons using the Bonferroni method. SB: spontaneous breathing, MV: mechanical ventilation, BALF: broncho-alveolar lavage fluid, IL: interleukin, PMN: polymorphonuclear leukocytes, S.p.: Streptococcus pneumoniae, SD: standard deviation.

In addition, according to our *ex vivo* assay which aimed at assessing the chemoattractive properties of BALF towards human PMNs, MV mildly decreased pulmonary migration of these cells in infected animals, but was shy of statistical significance (46 [22–59] vs 73 [52–76] % of attracted PMNs, p = 0.09) (Fig. 4).

### Pulmonary mitochondrial DNA levels and integrity dramatically decrease during pneumococcal pneumonia

**Figure 5:**
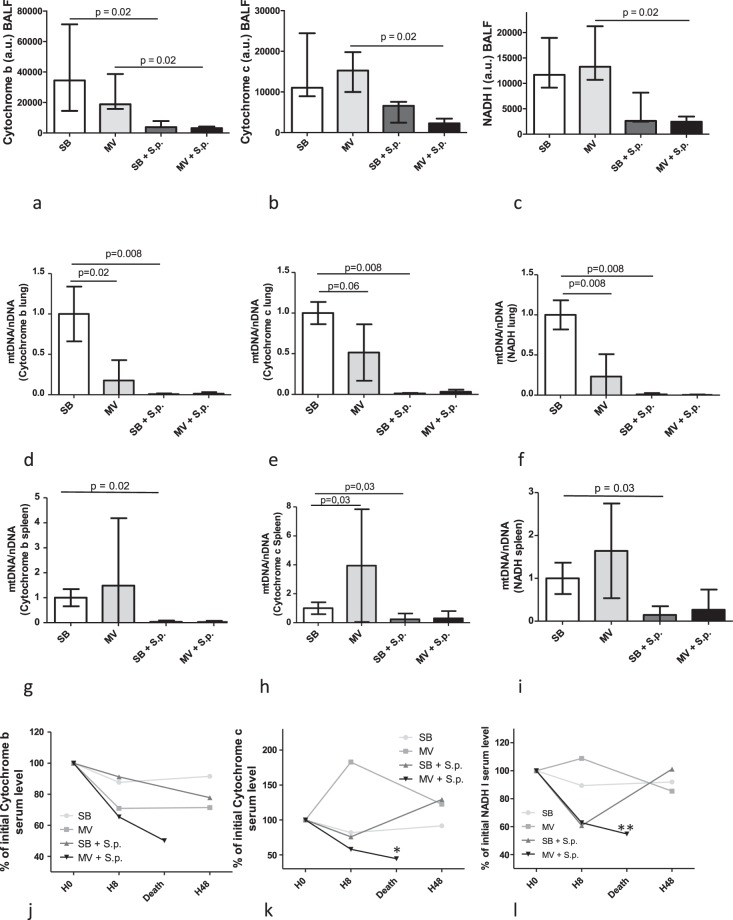
Mitochondrial DNA levels in broncho-alveolar lavage fluid, lung tissue, spleen and plasma in spontaneously breathing or mechanically ventilated rabbits with or without Streptococcus pneumoniae pneumonia. Median (IQR) mitochondrial DNA levels (Cytochrome b [a], Cytochrome c [b] and NADH I [c]) were measured in BALF, 48 hours (or at the time of death if earlier) after tracheal instillation of saline (controls) or 5.108 CFU of Streptococcus pneumoniae in spontaneously breathing rabbits or mechanically ventilated rabbits. Mitochondrial DNA levels were also measured in the lung (Cytochrome b [d], Cytochrome c [e] and NADH I [f]) and in the spleen (Cytochrome b [g], Cytochrome c [h] and NADH I [i]). Plasma concentrations (Cytochrome b [j], Cytochrome c [k] and NADH I [l]) were measured at baseline, 8 or 48 hours (or at the time of death if earlier) after saline or bacterial challenge. Results are expressed as percentage of baseline level. The Kruskall Wallis test and the Mann-Whitney U test were used successively for all intergroup comparisons and followed by post hoc corrections for multiple comparisons using the Bonferroni method. * denotes p = 0.05 and ** denotes p = 0.08 between infected SB and MV rabbits at H48 or time of death, respectively. SB: spontaneous breathing, MV: mechanical ventilation, BALF: broncho-alveolar lavage fluid, NADH: Nicotinamide adenine dinucleotide, PMN: polymorphonuclear leukocytes, S.p.: Streptococcus pneumonia, IQR: interquartile range.

**Figure 6:**
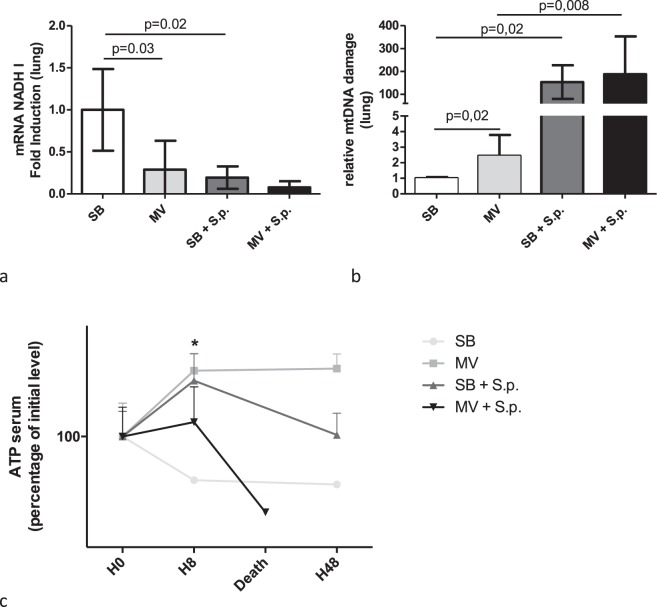
Pulmonary mitochondrial gene transcription, ATP plasma level and deleted mitochondrial DNA proportion in spontaneously breathing or mechanically ventilated rabbits with or without Streptococcus pneumoniae pneumonia. Pulmonary mitochondrial NADH-I mRNA was measured by reverse transcriptase-PCR (RT-qPCR), and normalized to GAPDH mRNA, 48 hours (or at the time of death if earlier) after tracheal instillation of saline (controls) or 5.108 CFU of Streptococcus pneumoniae in spontaneously breathing or mechanically ventilated rabbits [a]. Pulmonary mtDNA damage was assessed by quantifying the 4977bp deletion in mtDNA by PCR, and normalized to the mitochondrial NADH I gene [b]. The level of plasma ATP was measured at baseline, 8 or 48 hours (or at the time of death if earlier) after challenge [c]. The Kruskall Wallis test and the Mann-Whitney U test were used successively for all intergroup comparisons and followed by post hoc corrections for multiple comparisons using the Bonferroni method.ATP: adenosine triphosphate; SB: spontaneous breathing, MV: mechanical ventilation, RNA: ribonucleic acid, GAPDH: glyceraldehyde-3-phosphate dehydrogenase, NADH: Nicotinamide adenine dinucleotide, PCR: polymerase chain reaction, S.p.: Streptococcus pneumoniae.

Adverse MV alone did not increase the amount of mtDNA released within the airway (i.e., extracellular mtDNA) as reflected by BALF concentrations (Fig. 5). In contrast, mtDNA tissue content (i.e., intracellular mtDNA) as well as the corresponding genes expression in the lung, reflecting mtDNA amount and integrity as well, were significantly decreased 48 hours after the onset of adverse MV (Fig. 6). In addition, the proportion of deleted mtDNA (i.e., damaged mtDNA) was increased by adverse MV.

In SB animals, mtDNA levels were found to be significantly lower in the BALF of infected rabbits than in the uninfected ones (Cytochrome b: 3792 [3700–7777] vs. 34528 [14479–71283] a.u., respectively; p = 0.02) (Fig. 5). The same results were obtained for both Cytochrome c and NADH I genes (p = 0.02), as well as in MV rabbits. We found also significantly smaller amounts of intracellular mtDNA within the lung in infected rabbits as compared to uninfected ones. Moreover, the expression of both NADH I and Cytochrome b genes was significantly decreased within the infected lung (Fig. 6). The same results were obtained in both SB and MV animals.

In addition, the proportion of deleted mtDNA was far greater in the infected animals than in the uninfected ones, regardless of ventilation.

Since *S. pneumoniae* usually release one endonuclease (i.e., EndA) likely to hydrolyze circulating bacterial DNA, we hypothesized that EndA could have damaged mtDNA within the tissues of infected animals. However, there was no substantial loss of mtDNA detection *in vitro* after co-incubation with increasing concentrations of *S. pneumoniae*, emphasizing thereby the validity of our findings (Fig. S2).

### Circulating mitochondrial DNA levels decrease during *Streptococcus pneumoniae* pneumonia and return to baseline values only in the spontaneously breathing animals

Mitochondrial density and byproducts were also evaluated within the systemic compartment of infected animals. Thus, mtDNA concentrations were found to be significantly lower in the spleen of infected animals (i.e., intracellular mtDNA), whenever they were mechanically ventilated or not, whereas MV alone had no impact (Fig. 5).

Accordingly, plasma mtDNA level (i.e., extracellular mtDNA) decreased 8 hours after the onset of pneumonia in both infected groups. However, it rebounded and returned to baseline values 48 hours later in SB animals, whilst mtDNA concentrations remained low in MV animals, until death occurred.

In line with those findings, ATP plasma levels were found to be significantly lower in the animals subjected to MV than in their SB counterparts, as early as the 8^th^ hour after bacterial challenge (Fig. 6).

**Figure 7:**
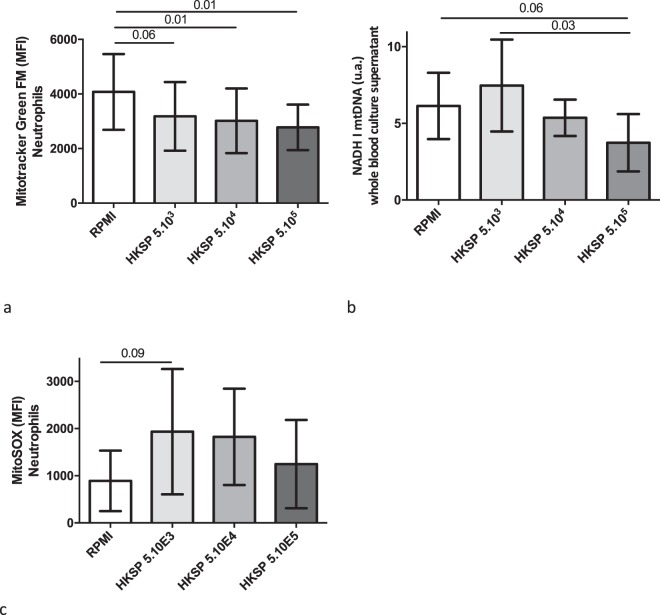
Mitochondrial homeostasis disturbance in whole blood stimulated ex vivo by Heat Killed Streptococcus pneumoniae (HKSP). (**a**) Neutrophil mitochondrial density assessed by flow cytometry with Mitotracker Green FM, and expressed as a median fluorescence intensity (MFI), in whole blood stimulated 16 hours with different concentrations of HKSP (5×103, 5×104, 5×105 bacteria/mL) or unstimulated (RPMI). Experiments were performed eight times. Data represent mean ± SD, n=8 per condition. Normality distribution was verified with D’Agostino-Pearson normality test and t-test was used for all intergroup comparisons. A statistically significant correlation was demonstrated by the Spearmann test (r = −0.34; p = 0.03). (**b**) Mitochondrial DNA NADH I level in cell culture supernatant after whole blood stimulation by HKSP. t-test was used for all intergroup comparisons. A statistically significant correlation was demonstrated by the Spearmann test (r = −0.34; p = 0.03). (**c**) Mitochondrial ROS assessed by flow cytometry with MitoSOX expressed as a median fluorescence intensity (MFI) in the experimental conditions described above. Data represent mean ± SD, n=8 per condition. t-test was used for all intergroup comparisons.DNA: deoxyribonucleic acid, HKSP: Heat Killed Streptococcus pneumoniae, MFI: Median fluorescence intensity, NADH: nicotinamide adenine dinucleotide dehydrogenase, RPMI: Roswell Park Memorial Institute medium, SD: standard deviation.

We conducted *ex vivo* stimulation assays in an attempt to decipher what a decrease in mtDNA plasma concentrations resulting from bacterial insult meant in terms of mitochondrial density within the circulating immune cells (Fig. 7). Interestingly, when rabbit whole blood was submitted to heat-killed *S. pneumoniae*, greater was the bacteria number, lower was the PMNs mitochondrial density (r = −0.34; p = 0.03) in parallel with a drop of the plasma mtDNA concentration (r = −0.42; p = 0.02). In addition, the release of mtROS by PMNs was then blunted, but we failed to show any statistically significant correlation.

### Mitochondrial depletion resulting from *Streptococcus pneumoniae* pneumonia may be related to defective biogenesis

**Figure 8:**
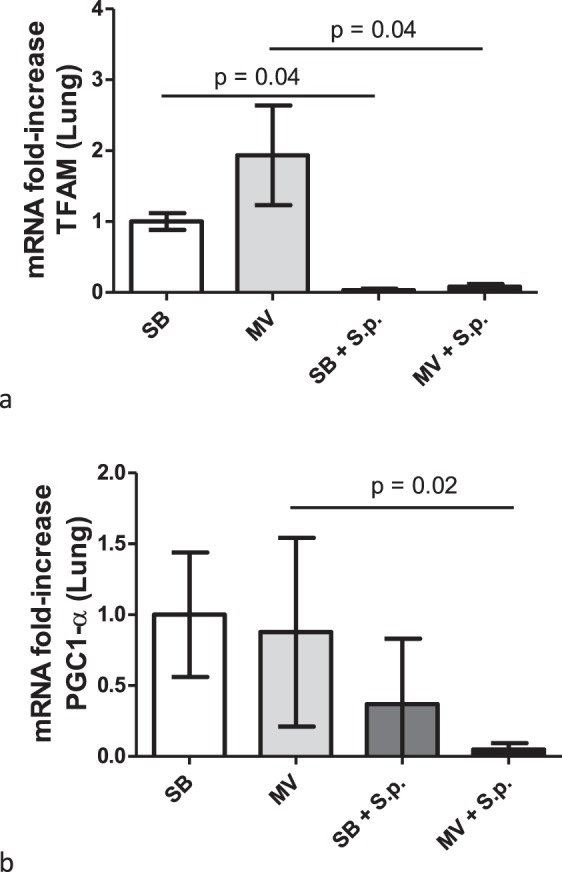
Lung mitochondrial biogenesis evaluation in spontaneously breathing or mechanically ventilated rabbits with or without Streptococcus pneumoniae pneumonia. Relative mRNA copy numbers of mitochondrial biogenesis genes (PGC1-α and TFAM) within the lung were measured by the reverse transcriptase-polymerase chain reaction. All values are shown as the fold increase compared with uninfected spontaneous breathing rabbits - value set to 1. The Kruskall Wallis test and the Mann-Whitney U test were used successively for all intergroup comparisons and followed by post hoc corrections for multiple comparisons using the Bonferroni method.

Since mitochondrial density is thought to depend on mitochondrial biogenesis, we investigated the impact of MV and *S. pneumoniae* pneumonia on these pathways within the lung. Hence, we observed within the infected lung a down-regulation of the main regulator of mitochondrial biogenesis PGC1-α, as well as of the key regulator of mitochondrial genome copy number TFAM, especially in the MV animals (Fig. 8).

## Discussion

As supported by our data, MV dramatically worsens the outcome of rabbits with *S. pneumoniae* pneumonia. Accordingly, the mortality rate reached 100% in the MV group whereas all the infected SB animals survived 48 hours after bacterial challenge. The substantially higher incidence of systemic dissemination in the ventilated rabbits, leading to overwhelming and unsolved systemic inflammation, could account for these differences, thus reflecting clinical observations^28^. The lack of efficacy of the lung immune response might explain this increased pulmonary-to-systemic translocation rate since MV significantly decreased bacterial clearance in the lungs. Accordingly, we showed that PMN chemotaxis was decreased within the lung of infected animals subjected to MV independently from IL-8 production, together with the release of larger amounts of IL-10, a powerful anti-inflammatory cytokine. Given the mitochondrial involvement in the regulation of the immune response, we hypothesized that significant derangements of their homeostasis could contribute to the highly harmful effect of MV in the pneumonia setting. Indeed, our findings indicate that MV alone was associated with a significant decrease in mitochondrial density within the lung. Moreover, *S. pneumoniae* infection itself led to an obvious drop of the mitochondrial content in both pulmonary and systemic compartments, especially in the animals under MV. Thus, the PMNs recruitment deficiency measured in the ventilated lung could be subsequent to mitochondrial depletion, given the chemoattractant properties of fMLP. In addition, it is worth noting that circulating mtDNA levels fell continuously until death in the infected MV rabbits whereas they returned to baseline values in their SB counterparts. Assuming that mtDNA concentrations reflected mitochondrial density within immune cells, as suggested by our *ex vivo* findings as well as by the concomitant decrease of plasma ATP, one could consider that antimicrobial defenses were also impaired within the bloodstream. Thus, mitochondrial depletion could account for the host failure to clear circulating bacteria, leading in turn to higher levels of systemic inflammation^24,29^. Mitochondrial depletion could result from a decreased biogenesis as reflected by the down regulation of both PGC1α and TFAM genes. However, it remains difficult to delineate the respective effects of MV and infection on the reported mitochondrial disorders. Obviously, the effect of MV on pulmonary mitochondria density was hidden by the effect of infection when the animals were concomitantly submitted to both. Nevertheless, it is worth noting that: (i) down regulation of PGC1 gene expression within the lung was more pronounced in the ventilated and infected animals; (ii) constantly decreasing circulating mtDNA concentrations were only seen in the MV animals with pneumonia. Altogether, our findings suggest that MV does contribute to the poor outcome of rabbits with pneumonia by enhancing mitochondrial disturbances.

Mitochondrial involvement during the host response to bacterial insult is paramount^30^. The clinical relevance of those findings has risen since some authors showed that on one hand, deep metabolic disturbances occurred in human cells, whereas on the other hand, more recently, others found that plasma concentrations of mtDNA were increased in both trauma and septic patients^14,22,23,31,32^. This latter finding is however controversial in sepsis since mtDNA depletion in blood and mononuclear cells was reported elsewhere in patients with sepsis and correlated with disease severity^24,29^. Similarly, a negative correlation between circulating cell-free mtDNA levels and all-cause mortality was found in a large cohort of patients, and in mechanically ventilated critically ill patients as well^25,33^. However, the case mix of patients, the variability of the studied mitochondrial genes, as well as the various measurement methods used for mtDNA detection, may account for these differences.

To the best of our knowledge, experimental data published so far regarding the involvement of mitochondria in VILI and bacterial pneumonia are even sparser and conflicting^18^. Thus, mtDNA infusion is likely to cause lung injury in a rodent model mimicking the human polytrauma setting, while lung stretch-related mtDNA alterations promote VILI in mice submitted to adverse MV^14,34^. In contrast, as we observed in our long-term adverse MV rabbit model, levels of mtDNA in BALF were not increased in mice ventilated for a short time with either low (8 mL/kg) or high (32 mL/kg) tidal volumes^35^. The existing discrepancy between the findings obtained in animals submitted to lung stretch as compared to other trauma models could be related to the extent of tissue damage caused by mechanical stress exposure. In the setting of bacterial pneumonia, it has been shown that rising mtDNA levels were detected within isolated perfused rat lungs infected with *Pseudomonas aeruginosa*, thus accounting for the formation of tissue edema^36^. Likewise, it has been recently reported *in vitro* that *S. pneumoniae* was likely to drive mitochondria disturbances leading to the release of mtDNA by infected alveolar cells through pneumolysin secretion, one of its most important toxins^37^. However, the animal model used in the present study has several advantages including the ability to assess long-term adverse MV and survival in animals with pneumonia.

In the context of sepsis, the link between mitochondrial homeostasis regarding the host immune response remains unsettled. Some authors reported that, in mice with *S. aureus* peritonitis, a significant decrease in liver concentrations of mtDNA occurred during the first two days, followed by complete restoration of mitochondrial density in survivors only, resulting from mitochondrial biogenesis enhancement^38^. Similar data were obtained in the muscles of mice submitted to CLP^39^. Our findings suggest an impairment of mitochondrial biogenesis within the lung of infected animals, accounting for depletion of protracted organelles. This finding paves the way for further therapeutic prospects, since increased mitochondrial biogenesis has been associated with survival in sepsis^40^.

The pathophysiological link between such mitochondrial dysfunctions and the reduced efficacy of the host immune response is still an unsolved issue. Accordingly, mtDNA is required for NLRP3 inflammasome activation. Macrophages lacking mitochondria are thereby less likely to produce IL-1ß, since NLRP3 is required for the cleavage of pro-IL-1ß into bioactive IL-1ß, thus hampering their ability to clear pathogens^16^. Indeed, the beneficial effects of treatment with CpG ODN, a powerful TLR9-agonist, on bacterial clearance and mortality were demonstrated in animal models of *S. pneumoniae* and *S. aureus* pneumonia. These effects were achieved by restoring immune functions^41,42^. However, such findings are controversial since others have shown that a single mtDNA injection caused profound, TLR9-dependent immunosuppression in mice^32^.

Several limitations should be mentioned. First, the MV without PEEP we used could be considered not clinically relevant. However, it has recently been reported that such settings are still used, especially in the operating room^43^. In addition, it is known that lung injury is heterogeneous in ARDS patients. As a result, poorly aerated areas of the lung usually coexist with overstretched ones, even if “low-V_T_” is applied, as shown in human studies^44^. Moreover, our model is likely to illustrate the respective effects of adverse MV (i.e. VILI features) and pneumococcal infection. We should also acknowledge that by using SB animals as controls, lung stretch was not the only factor evaluated. Actually, one cannot exclude the possibility that other mechanisms, such as impaired airway drainage subsequent to tracheal intubation, a prolonged supine position and general anesthesia could account, at least in part, for the lower bacterial clearance rate we report here. However, we have previously published data showing that when mild-stretch MV was compared with low-stretch MV in a Gram-negative bacteria VAP model, the latter strategy was likely to favor the host regarding this point^8^. We cannot exclude that mitochondrial density was underestimated by mtDNA concentrations measurement since oxidized DNA as found in stressed organelles was not necessarily amplified. However, such a hypothesis is unlikely since 3 different mitochondrial genes were amplified with the same results. Finally, the fact that animals were not given antibiotics made any translation to clinical practice questionable. Further experimental studies are therefore needed.

In conclusion, MV impaired lung bacterial clearance and promoted pulmonary-to-systemic translocation in our model. As a result, higher levels of blood inflammation and increased mortality rates were observed. Simultaneously, these rabbits showed obvious signs of mitochondrial depletion in both the lungs and the overall system, together with evidence for deficient biogenesis. Altogether, although speculative, these data suggest that mitochondrial homeostasis is impaired in the setting of *S. pneumoniae* pneumonia, especially if the mechanical stretch resulting from MV is combined with the onset of infection, thereby opening new therapeutic avenues.

## Methods

### Animals

Male New Zealand White rabbits (3.0 to 3.3 kg) were bred in the University of Burgundy animal facility (Dijon, France). Animal use was approved by the local veterinary committee (i.e., Comité d’éthique de l’expérimentation animale Grand campus Dijon [C2EA Grand campus Dijon] n°105) and experiments were performed according to European laws for animal experimentation (European regulation on Animal Welfare).

### Experimental Design

We conducted a prospective randomized animal study. Two sets of experiments were conducted, including uninfected and infected animals, respectively. Within each set, animals were randomized by drawing lots into spontaneously breathing or mechanical ventilation groups. The following groups were obtained: uninfected controls (SB [n = 5], or MV [n = 5]), infected (SB [n = 5] + S.p., or MV + S.p. [n = 5]) groups.

### Mechanical Ventilation Model

The animals were intubated as previously described^45^. Briefly, under general anesthesia provided by ketamine 20 mg/Kg (Panpharma, France) and xylazine 1,5 mg/Kg (Rompun®, Bayer, Germany), a cuff tube of 2.5 mm (Mallinckrodt**™**, Covidien®, U.S.A.) was orally introduced into the trachea under view control. The animal was put in the supine position and connected to a volume-controlled respirator (Servo ventilator 900 C, Siemens®, Germany) (12 mL/kg of tidal volume with zero end-expiratory pressure [ZEEP], a respiratory rate of 30 bpm and an 0.5 inspired fraction of O_2_), since it has been shown that VILI features are obtained with such settings^46,47^. Only ventilated rabbits were kept anesthetized and paralyzed throughout the experiment with midazolam (0.2 mg/Kg/h) (Hypnovel®, Roche, Switzerland) and cisatracurium besilate (0.8 mg/Kg/h) (Nimbex®, GlaxoSmithKline, U.K). The animals were placed on a heating blanket, and isotonic serum was infused. Non-invasive monitoring was used to monitor heart rate (Hewlett Packard 78353B Monitor). Arterial blood lactate and gases were measured just after intubation to ascertain the safety of our “adverse” MV and at 48 hours (or immediately before death, when bradycardia prior to asystole occurred).

### Experimental Pneumonia Induction

The pneumococcal clinical strain 16089 (9V serotype) was used (kindly provided the Centre National de Référence des Pneumocoques, France). Bacteria were grown in 5% CO2 in brain heart infusion (BHI) broth (BioMérieux, Marcy l’Etoile, France). Before each experiment, bacteria from one frozen aliquot was cultured on agar plates and incubated for 24 h at 37 °C. Twenty-five to 30 colonies were inoculated into 9 ml of BHI broth (BioMérieux, Marcy l’Etoile, France) for 6 h at 37 °C, and then cultured on agar plates for 18 h at 37 °C in an anaerobic atmosphere. This culture was diluted in physiologic saline to obtain a final inoculum of 8.5 log_10_ colony-forming units (CFU)/ml in 0.5 ml of saline, according to optical density measurements in reference to a standard curve and confirmed by culture. Pneumonia was induced as previously described^48^, by endobronchial challenge with 0.5 ml of this freshly calibrated bacterial inoculum in either SB or MV animals.

### Pneumonia Evaluation

Forty-eight hours after pneumonia induction, the animals were euthanized by pentothal overdose following ketamine-xylazine injection as described above, and exsanguinated. However, if death occurred earlier (by asystole), animals were autopsied within the 2 minutes following exsanguination^45^. Spontaneously breathing animals were euthanized if necessary (clinical signs of upcoming death), whereas the MV rabbits were already under general anesthesia. The lungs were removed via thoracotomy and lung injury evaluation was based on a macroscopic score and microscopic examination. For each lower lobe, a sample measuring 1 cm^3^ focused on a macroscopic lesion was excised, fixed in 10% buffered formalin, and embedded in paraffin. Hematoxylin-eosin staining was applied. Each specimen was graded using a score ranging from 0 to 3, based on the degree of neutrophil infiltration, hemorrhage, and edema^49^.

### Quantitative Bacteriology in Infected Lung and Spleen

Lungs and spleen from each animal were removed and homogenized. Bacteria were counted in a sample of this crude homogenate by plating 10-fold dilutions on sheep blood agar and incubating the plates for 24 h at 37 °C. For each rabbit, the mean concentration was calculated (e.g., mean concentration = Σ [organ concentration × organ weight]/Σ organ weights), and adjusted for the dilution.

### Assessment of Inflammation

Blood samples were obtained just before experiment onset (H0), 8 hours later (H8) and at H48 (or just before death). Blood, lung and spleen concentrations of IL-8, IL-1ß, IL-10 and TNF-α were assessed by enzyme-linked immunosorbent assay (ELISA) (Euromedex). Lung and spleen pieces were taken and RNA was extracted using the RNA GenElute kit (Sigma). Complementary DNA (cDNA) was obtained by reverse transcription using random primers, RNAsin treatment, and ImProm II reverse transcriptase (Promega, Madison, WI). Quantitative PCR was then performed using the IQ5 thermocycler (Biorad, Hercules, CA) and the IQ^TM^ Syber Green Supermix (Biorad) and rabbit-specific primers, designed using Primer3 software (version 0.4.0), and the rabbit (*Oryctolagus cuniculus*) sequence database. Melting curves were performed to ensure the presence of a single amplicon. The primers sequences for Gapdh, Il-8, Il-1β, were reported in Table S1. The results are expressed as the fold induction using the ΔCt method since the spontaneously breathing (SB) animals were always considered the baseline condition.

### Mitochondrial DNA, mRNA and Mitochondrial Biogenesis Assessment

Mitochondrial DNA was measured in plasma and BALF (circulating cell-free mtDNA), as well as in lung and spleen tissue (reflecting mitochondrial density). Quantitative PCR was used to measure levels of mtDNA using specific PCR primers for rabbit cytochrome B, cytochrome C oxidase III, and NADH I^14^ (Table S1). Primers were designed using the *Oryctolagus cuniculus* mitochondrion complete genome NCB1 reference sequence (NC-001913.1), synthesized by Microsynth (Balgach, Switzerland), and had no significant homology with sequences from rabbit genomic DNA (Blast® site, http://blast.ncbi.nlm.nih.gov).

DNA was isolated from blood and BALF, using the QIAamp DNA Mini Kit (Qiagen, Valencia, CA, USA), with a final volume of 200 µl of DNA resuspended in elution buffer. Quantitative PCR was performed with one-tenth or one-hundredth dilutions of the final product, compared with a standard curve of rabbit mtDNA to quantify the amount of amplified mtDNA, and expressed as arbitrary units. Melting curves were performed to ascertain the amplification of a single amplicon. Rabbit mtDNA was isolated from peripheral blood mononuclear cells of healthy rabbits using the mitochondrial isolation kit for cultured cells from ThermoScientific (Rockford, IL, USA). To further ensure that cytochrome b, c and NADH-I primers were specific to mtDNA and did not amplify bacterial DNA sequences, we performed real-time PCR using DNA isolated from our *S. pneumoniae* strain. In order to assess the mitochondrial density within the lung and spleen parenchyma, mtDNA levels were then measured in tissue homogenates. Total cellular DNA was extracted from frozen lung and spleen with the DNeasy blood and Tissue kit (Qiagen, Valencia, CA, USA). The mtDNA copy number was obtained by real-time PCR as previously described and normalized to a nuclear house-keeping gene (i.e., GAPDH) expression, in order to ensure that these concentrations were not related to the number of live cells within the tissue sample^50^. In addition, mitochondrial genes expression was also evaluated through the corresponding mRNA quantification by qPCR, as a reflect of mitochondrial proliferation and function. Mitochondrial DNA damage was assessed by quantifying a large base pair deletion along the major arch of the mitochondrial genome by quantitative PCR. A common 4977 bp deletion in mtDNA was quantified by quantitative PCR using primers flanking the deletion and normalized to the mitochondrial NADH I gene.

Mitochondrial biogenesis was assessed through the measurement of PGC1-α and TFAM gene expression, in the lung tissue, by qPCR. The corresponding primers sequences for are reported in Table S1.

### Neutrophils chemotaxis evaluation

Chemotaxis of human neutrophils induced by rabbits’ BALF was measured using a modified Boyden chamber^51^. For each experiment, serial dilutions of prototypical chemotactic factors, such as human IL-8 (Merck-Serono, Geneva, Switzerland) and bacterial/mitochondrial fMLP (Sigma, St. Louis, MO, USA) served as a control for the maximal rate of neutrophil chemotaxis. Human neutrophils from healthy volunteers were isolated using a Ficoll-Paque^TM^ (GE Healthcare Bio-Sciences AB, Uppsala, Sweden) gradient and seeded into the upper well. Different samples of BALF were tested. Results were expressed as % of neutrophils from the upper well migrating across the filter to the lower well after 90 min. Neutrophils in the lower well were detected with the DraQ5^TM^ dye (Biostatus), which stains neutrophil DNA, and counted using the Applied Biosystems 8200 Cellular Detection System (Life Technologies, Switzerland) in comparison with a known concentration of neutrophils as standard.

### Circulating ATP concentrations measurement

Plasma ATP was quantified using the ATP bioluminescence assay kit CLS II with a detection range of 10^−11^–10^−6^ M (Roche Applied Science, Mannheim, Germany). A standard curve was performed with purified ATP.

### *Ex vivo* whole blood stimulation assays

Fresh heparinized blood from healthy male rabbits was obtained by venipuncture and diluted 1:5 with RPMI 1640 medium (GibcoTM Life Technologies, Saint Aubin, France), supplemented with glutamine 2 mM (Gibco), and Fetal Bovine Serum 5% (Sigma). Blood was stimulated with 5 × 10^3^, 5 × 10^4^ or 5 × 10^5^ bacteria/mL heat-killed *S. pneumoniae* (HKSP) or left unstimulated. Viable bacteria were not used due their proliferation during the experiment and the need to control the inoculum size. HKSP were prepared by exposing a calibrated inoculum of *S. pneumoniae* (strain 16089), to 95 °C for 15 min. After 16 hours of incubation at 37 °C, diluted blood was centrifuged for 10 min at 500 g. Cell culture supernatant was removed and cell pellet used for mitochondrial staining after removing red blood cells with a 6 min red blood cells lysis. The supernatant was centrifuged for another 5 min at 5,000 g and kept frozen at −80 °C before use.

### Mitochondrial labeling

For measuring the mitochondrial mass and mitochondrial ROS production, cells were labeled with respectively Mitotracker green FM and MitoSOX probes (Thermo), according to the manufacturer’s instructions. In brief, cells were rinsed with PBS and resuspended in PBS-0.5% BSA before staining with Mitotracker green FM, or MitoSOX at a final concentration of respectively 150 nM and 5 nM, and incubated at 37 °C, 5% CO2, during 20 min in dark. The concentration of mitochondrial dye used was selected by titrating with different concentrations and the same concentration was used throughout all experiments. Cells were then washed with 5 ml PBS and then centrifuged for 5 min at 500 g. Cell viability was determined by Zombie Violet staining (Biolegend). Cells were then washed with 5 ml PBS, centrifuged for 5 min at 500 g and scrapped in 300 µl PBS 0.5% BSA. Data were acquired on a BD LSRFortessa^TM^ cytometer and analyzed using BD FACSDIVA (BD Biosciences, San Jose, CA) and FlowJo (TreeStar, Ashland, OR) software. Neutrophils were gated with SSC/FSC characteristics and doublets and dead cells were excluded. Results are expressed as a geometric median fluorescence intensity (MFI) of the mitochondrial labelling of the viable cells. Experiments were performed eight times.

Mitochondrial DNA concentrations were measured in the supernatant of whole blood stimulations assay according the method described above.

### *In vitro* analysis to test mtDNA hydrolysis by *Streptococcus pneumoniae*

A defined quantity of mtDNA (3 ng) was incubated for 6 hours at 37 °C in an anaerobic atmosphere with seven different concentrations of *S. pneumoniae* inoculum diluted to 1/10th (7 to 1 log_10_ CFU/mL), a negative (absence of *S. pneumoniae*) and a positive control (RQ1 Dnase (Promega, Madison, WI). DNA extraction and PCR were performed as described above for both cytochrome b and NADH genes. Experiments were performed four times.

### Statistical analysis

Data are presented as medians (IQR). Group sizes were based on previous experiments in which statistically significant differences regarding, for instance, inflammatory mediator levels were achieved with similar numbers of animals^46,47^. The Kruskall Wallis test was used for all intergroup comparisons of continuous variables. If the result was statistically significant, the Mann Whitney *U* test (or the Wilcoxon test if appropriate) was performed in order to compare the two groups, unless otherwise stated. Post hoc corrections for multiple comparisons were then applied using the Bonferroni method. Regarding *ex vivo* experimental data, correlations between *S. pneumoniae* inoculum concentrations and some mitochondrial parameters (i.e., mitochondrial DNA concentrations or others) were analyzed with the Spearman test. The cumulative probability of progression to death was compared between groups using the Kaplan-Meier method and the log-rank test. All tests were two-tailed. A *p* value lower than 0.05 was considered statistically significant. Data were analyzed with Prism software (GraphPad Prism®, San Diego California, USA).

### Availability of supporting data

All the data are available on demand.

## Electronic supplementary material

Supplementary dataset 1
