# Supplementary material for: Mechanical ventilation and Streptococcus pneumoniae pneumonia alter mitochondrial homeostasis
Source: Sci Rep. 2018 Aug 6;8:11718. doi: 10.1038/s41598-018-30226-x (PMC6078986; doi:10.1038/s41598-018-30226-x)
Supplement: Supplementary file 1 — Supplementary dataset 1 [file 41598_2018_30226_MOESM1_ESM.docx]

**Mechanical ventilation and *Streptococcus pneumoniae* pneumonia**

**alter mitochondria homeostasis**

Mathieu Blot, MD^1,2^

[mathieu.blot@chu-dijon.fr](mailto:Mathieu.blot@chu-dijon.fr)

Laure-Anne Pauchard, PhD^1^

[laureanne.pauchard@gmail.com](mailto:laureanne.pauchard@gmail.com)

Irène Dunn, PhD^3^

irene.dunn-siegrist@unige.ch

Jennifer Donze, MS^1^

jennifer.donze@hotmail.fr

Stéphanie Malnuit, MS^1^

steph.malnuit@hotmail.fr

Chloé Rebaud^1^

chloerebaud@hotmail.fr

Delphine Croisier, Pharm, PhD^4^

[delphine.croisier@vivexia.fr](mailto:Delphine.croisier@vivexia.fr)

Lionel Piroth MD, PhD^2^

lionel.piroth@chu-dijon.fr

Jérôme Pugin, MD, PhD^3^

Jerome.Pugin@unige.ch

Pierre-Emmanuel Charles, MD, PhD^1,5^

pierre-emmanuel.charles@chu-dijon.fr


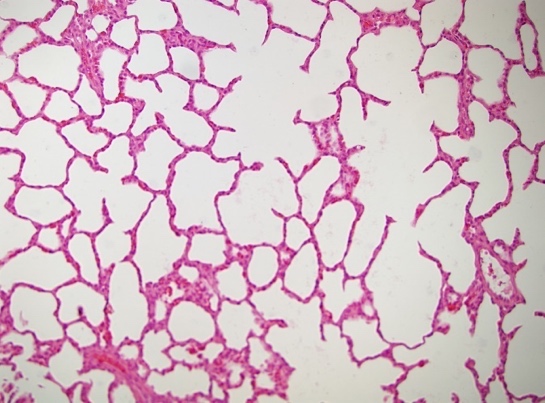

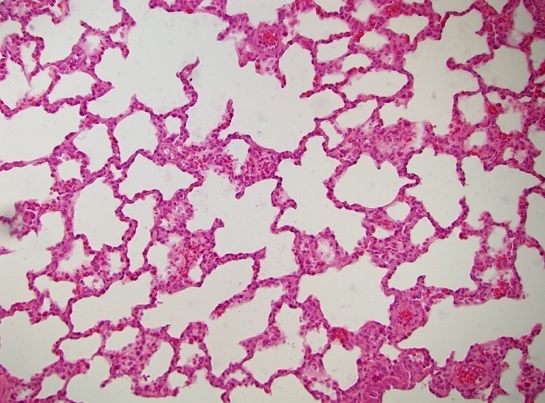

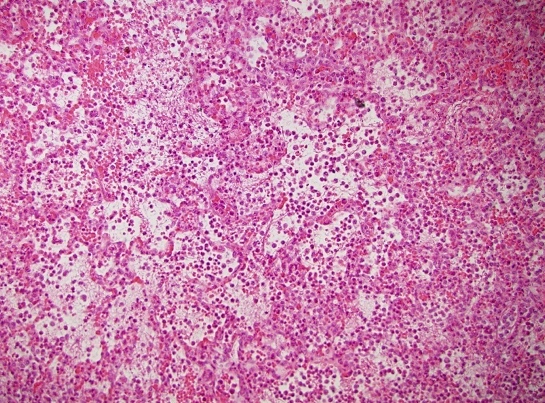

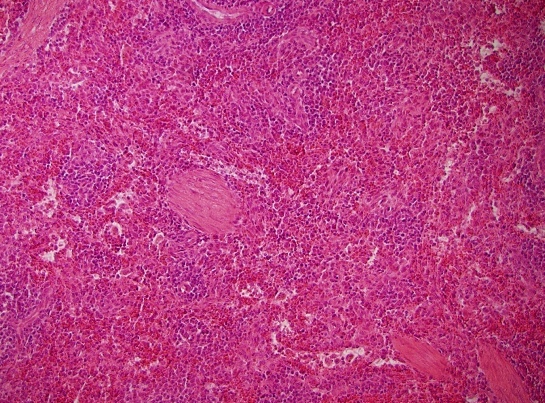


SB + *S.p.*

MV + *S.p.*

MV

SB

a

b

**Figure S1: Main features of lung injury in spontaneously breathing or mechanically ventilated rabbits with or without *Streptococcus pneumoniae* pneumonia.**

(A) Representative microphotographs of rabbit lungs fixed at the same transpulmonary pressure (hematoxylin and eosin x400) in various conditions: following tracheal instillation of saline (controls) or 8.5 log10 CFU of *Streptococcus pneumoniae* in spontaneously breathing or mechanically ventilated animals. (B) Histopathology score of Ventilator Induced Lung Injury ranging from 0 to 3, based on the degree of polymorphonuclear infiltration, hemorrhage, and edema in the interstitial and alveolar spaces, in spontaneous breathing or mechanically ventilated rabbits (B).

The two-tailed Mann-Whitney U test was used for all intergroup comparisons followed by appropriate corrections for multiple comparisons.

CFU: colony forming unit; MV: mechanical ventilation, SB: spontaneously breathing, *S.p*.: *Streptococcus pneumoniae*.

a b

**Figure S2. *in vitro* hydrolysis of mitochondrial DNA by *Streptococcus pneumoniae.***

Median concentrations (IQR) of mitochondrial DNA (Cytochrome b [A], NADH I [B]) were measured 6 hours after co-incubation with increasing concentrations of *Streptococcus pneumoniae*.

The two-tailed Mann-Whitney U test was used for all intergroup comparisons.

Deoxyribonucleic acid, Dnase: Desoxyribonuclease, NADH: nicotamide adenine dinucleotide hydrogen, PCR: polymerase chain reaction, IQR: interquartile range.

The two-tailed Mann-Whitney U test was used for all intergroup comparisons followed by appropriate corrections for multiple comparisons.

|  | forward | reverse |
| --- | --- | --- |
| GAPDH | 5’-ATG TTT GTG ATG GGC GTG AAC C- 3’ | 5’-CCC AGC ATC GAA GGT AGA GGA- 3’ |
| IL-8 | 5’-AAC CTT CCT GCT GCT TCT GA- 3’ | 5’-TCT GCA CCC ACT TTT TCC TTG- 3’ |
| IL-1β | 5’-CCT GTC CTG CGT GAT GAA AG- 3’ | 5’-GAC GGG CAT GTA CTC TGT CT- 3’ |
| cYT-b | 5’-CCA TCC TTG TTC TAG CCT TCA- 3’ | 5’-AAT GGT GAT GAA CGG GTG TT- 3’ |
| Cyt-C | 5'-GAA GGC AAT CGC AAA AAC AT- 3’ | 5'-ACG TGA AGA CCG TGA AAT CC- 3’ |
| NADH-I | 5' -GCC CCA ACC CTA GCT CTA AC- 3’ | 5'-GCT CGG AGA GCA CCA AAT AG- 3’ |
| PGC1-α | 5’ -TAG AGT CTT GGA GCT CCT- 3’ | 5’ -TCC TCT GAC CCC AGA CTC AC- 3’ |
| TFAM | 5’ -TAT AAG CTG AAC GAG GTC- 3’ | 5’ -ACA ACT ACC CAT ATT TAA AGC- 3’ |
| mtDNA_4977pb_ | 5’-CCTTACACTATTCCTCATCACC-3’ | 5’-TGTGGTCTTTGGAGTAGAAACC-3’ |

**Table S1: Polymerase chain reaction rabbit-specific primers sequences.**

Abbreviations: GAPDH: glyceraldehyde-3-phosphate dehydrogenase, IL: interleukin, Cyt: cytochrome, NADH: nicotamide adenine dinucleotide, ATG: autophagy-related, PGC1: peroxisome proliferator-activated receptor gamma coactivator 1, TFAM: mitochondrial transcription factor A.

|  | PaO_2_/FIO_2_ ratio | | Arterial lactate concentration (mmol/L) | | Arterial pH | |
| --- | --- | --- | --- | --- | --- | --- |
|  | H0 | H48 | H0 | H48 | H0 | H48 |
| MV | 453 (412-459) | 420 (409-437) | 1.4 (1-1.5) | 1.0 (0.8-1.3) | 7.49 (7.43-7.51) | 7.48 (7.47-7.49) |
| MV + S.p. | 413 (397-431) | 142 (78-215)* | 1.6 (1.4-1.9) | 6.5 (5.1-7.3)* | 7.53 (7.50-7.56) | 7.16 (7.14-7.26) |

**Table S2: Arterial blood oxygenation, pH and lactate concentration in rabbits subjected to**

**mechanical ventilation with or without *Streptococcus pneumoniae* pneumonia.**

Blood samples were obtained at baseline (H0), and after 48 hours of mechanical ventilation or

earlier if death occurred**.

Data are expressed as median (IQR).

*indicates p<0.05

The two-tailed Mann-Whitney U test was used for all intergroup comparisons.

**blood was drawn just before death, at the onset of bradycardia. SB: spontaneous breathing,

MV: mechanical ventilation, *S.p*.: *Streptococcus pneumoniae*, PaO2/FIO2 ratio: ratio of

arterial partial oxygen pressure /fraction of inspired oxygen.
